# PD07 Economic Analysis Of Visualase Laser Interstitial Thermal Therapy For Tumors In Australia

**DOI:** 10.1017/S0266462325101451

**Published:** 2025-12-29

**Authors:** Rashmi Joglekar, Gail Dechochai, Santwona Baidya

## Abstract

**Introduction:**

Visualase is a minimally invasive, MRI-guided system that uses laser interstitial thermal therapy (LITT) to treat patients with brain tumors and drug resistant epilepsy. Our objective was to perform an economic analysis of LITT compared with open cranial resection for patients with brain tumors, from the perspective of the Australian healthcare system and with consideration of equity for eligible patient populations.

**Methods:**

A literature review was carried out to gather international data on clinical outcomes of LITT and open resection for patients with brain tumors, as data in Australia are very limited. Cost data were sourced from 2024 Medicare and Pharmaceutical Benefits Scheme fees and hospital costs were sourced from the 2014 Australian Critical Care Resources Survey, costed at the 2023 consumer price index equivalent. A cost-consequence analysis was carried out to calculate the incremental cost per life year (LY) gained from the perspective of the Australian healthcare system. Subgroup analysis was carried out due to heterogeneity of the patient population.

**Results:**

Our analysis demonstrated that the cost per LY gained was AUD87,630 (USD56,318) for recurrent primary brain tumors, AUD24,630 (USD15,828) for recurrent brain metastases, and AUD15,948 (USD10,249) for radiation necrosis. Considering a willingness to pay threshold of approximately AUD50,000 per LY (USD32,131/LY), LITT could be cost effective for some patient groups.

The minimally invasive nature of LITT meant that hospital stay was reduced from an average of four days to one day, potentially increasing hospital throughput and reducing waiting lists. The model could be further refined with inputs from patients in the Australian setting and consideration of equity, when such data become available.

**Conclusions:**

From a payer perspective, LITT can be a cost-effective option in Australia for certain patient groups. The full economic value to society could be demonstrated by quantifying the utility of patient preference for minimally invasive procedures, and the benefits of patients and carers returning to school or work. Equity considerations or the rule of rescue may be applicable for eligible patient populations.

